# Three-Dimensional Printing Process for Musical Instruments: Sound Reflection Properties of Polymeric Materials for Enhanced Acoustical Performance

**DOI:** 10.3390/polym15092025

**Published:** 2023-04-24

**Authors:** Tomáš Zvoníček, Martin Vašina, Vladimír Pata, Petr Smolka

**Affiliations:** 1 Department of Physics and Materials Engineering, Faculty of Technology, Tomas Bata University in Zlín, Vavrečkova 5669, 760 01 Zlín, Czech Republic; 2 Department of Production Engineering, Faculty of Technology, Tomas Bata University in Zlín, Vavrečkova 5669, 760 01 Zlín, Czech Republic; 3 Centre of Polymer Systems, Tomas Bata University in Zlín, Třída Tomáše Bati 5678, 760 01 Zlín, Czech Republic

**Keywords:** sound reflection coefficient, musical instrument, fused deposition modeling, 3D printing, surface texture

## Abstract

Acoustical properties of various materials were analyzed in order to determine their potential for the utilization in the three-dimensional printing process of stringed musical instruments. Polylactic acid (PLA), polyethylene terephthalate with glycol modification (PET-G), and acrylonitrile styrene acrylate (ASA) filaments were studied in terms of sound reflection using the transfer function method. In addition, the surface geometry parameters (Sa, Sq, Sz, and Sdr) were measured, and their relation to the acoustic performance of three-dimensional-printed samples was investigated. It was found that a higher layer height, and thus a faster printing process, does not necessarily mean poor acoustical properties. The proposed methodology also proved to be a relatively easy and rapid way to test the acoustic performance of various materials and the effect of three-dimensional printing parameters to test such a combination at the very beginning of the production process.

## 1. Introduction

Acoustical properties of materials play a crucial role in the performance of musical instruments. For millennia, skilled artisans were searching for the best combination of materials to produce musical instruments with superb acoustics. Engineers, designers, architects, and others have focused on manufacturing products for musical performances, concert halls, churches, etc. The right design and material properties are crucial for quality sound distribution. Strings (stringed musical instruments) are arguably the largest family of instruments in the classic orchestra. They come in different sizes and variants. There is, however, one unifying aspect—the material used has historically almost exclusively been wood. The wood selection depends on the instrument, and can range from spruce to cottonwood, birch, maple, rosewood, ebony, etc. Various kinds of wood may be used for each part of a single instrument, based on the long-standing evidence (in other words, wood has often been chosen empirically) [1,2]. However, only wood of the highest quality (both acoustical and visual) has been utilized. As the yield of high-quality material per unit of material being harvested is relatively low, and the overall availability of quality lumber decreases for various reasons, the traditional manufacturers of musical instruments are facing the problem of an insufficient material supply. Alternative approaches have been examined to solve this issue. One is the chemical treatment of wood, while another can be lamination of wood with polymer-based materials, such as carbon fiber-reinforced composites (CFRCs) or the use of CFRCs alone for some parts of musical instruments. The main objective is to achieve the desired resonant properties of materials (the acoustic conversion efficiency), namely, a high Young’s modulus (elasticity) and low internal friction (energy loss) [3,4]. Until very recently, this has been the (almost) only goal in the production of musical instruments from alternative materials. With the advent of the COVID-19 pandemic, much has changed. The disease has had catastrophic effects worldwide, affecting almost every aspect of the everyday life. With the inevitable lockdowns in many countries, huge numbers of people were forced to stay home for a long period [5,6,7,8]. Many could continue to work using remote communication methods. However, this has been a problem for musicians, as they need day-to-day rehearsals so as not to lose their skills [9]. Namely, in highly populated areas with most people living in apartment blocks, rehearsing with a classical musical instrument very often means exposing neighbors to elevated sound levels (in other words, noise). Although electronic musical instruments were also available before the COVID-19 pandemic, it was largely a case of guitars and violins. With the spread of lockdowns, other musicians also began to search for an alternative to their wooden stringed instrument. The issue was not only a tolerable noise level, but also the price, as not everybody could afford to purchase another expensive instrument just for home-based rehearsals.

Additive manufacturing techniques, often referred to as 3D printing or additive manufacturing (the terms should not be used interchangeably, though), have revolutionized many industries in recent years. The examples include automotive, aviation, aerospace, and defense industries, medical and health industries, sport, leisure time activities, etc. Primarily, the main domain of 3D printing was prototyping, but it is slowly moving towards being accepted as a standard production method [10,11]. On the other hand, it retains the qualities for which it was originally valued—low price at low volumes (as opposed to, for example, injection molding) and a high level of customization. As such, the 3D printing technique seems to be an ideal candidate to produce small series of products that need to be tailored to the needs of the end customers, e.g., musical instruments. There are, however, inherent challenges related to additive techniques that need to be addressed, namely in the fused deposition modeling (FDM) technique, such as interlayer cohesion, overall mechanical properties, long-term stability (chemical and dimensional) [12,13,14], the effect of construction porosity (the printed models are hardly ever completely solid, they usually contain some percentage of voids), etc. [15,16,17,18]. All these factors can affect the sound absorption or reflection properties of the final product [17]. From the acoustical point of view, the reflection (absorption) of sound waves is affected by several parameters, such as surface shape, obstacles, and their spatial distribution, their distance, and their arrangement. Zulkifli [19,20] and Lee [21] reported that the acoustical absorption of multilayer materials is better with perforated plates backed with air gaps. In the study by Cucharero et al. [22], the authors showed how to measure angle-dependent absorption coefficients. The results confirmed that materials have a different behavior in response to sound waves approaching from different directions. Other studies describe specific factors, such as material thickness, density, particle sizes, and specific excitation frequencies, that affect the final properties of a sample [23,24].

**Figure 1:**
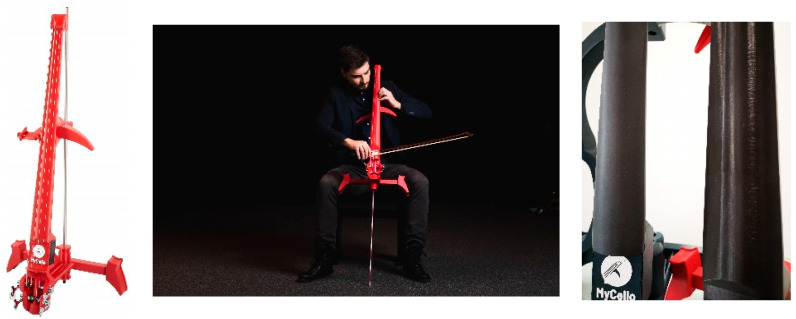
Electric violoncello produced by the FDM additive manufacturing technique [27].

The acoustics of materials plays an important role in the production of musical instruments [25,26]. Musical instruments manufactured by 3D printers have several advantages compared to traditional manufacturing methods. As stated above, they can be cheaper and better customized to the needs of an individual musician. Their performance does not need to meet that achieved with the original (wooden) instruments, but it must be reasonably high. Affordability and other qualities of the 3D-printed instrument, such as the ability to play quietly (not disturbing neighbors), can balance some of the construction imperfections. There are already some companies producing 3D-printed string instruments on a commercial basis. There has been high demand for an affordable and customizable electric violoncello among professional musicians in the last years. Such products already exist, as shown in Figure 1. The combination of 3D printing, polymer materials, and rather high load (equivalent of 50 to 60 kg in a standard violoncello, depending on the strings used) necessitates a production process that retains a low cost (layer height, printing speed, infill density, and pattern), while also providing long-term stability and not compromising the acoustical properties.

The present study expands the research on the acoustic properties of materials produced by additive technology with distributed porous structures. It investigates the sound reflection properties of three different types of 3D-printed materials, namely polyethylene terephthalate with glycol modification (PET-G), acrylonitrile styrene acrylate (ASA), and polylactic acid (PLA). To our knowledge, no relevant studies examining the sound reflection properties of the above types of 3D-printed materials, considering the influence of several factors, such as their structural type, excitation frequency, and the surface quality, have been published, yet. The results presented in this paper could help in the development of affordable 3D-printed musical instruments that, with the help of sensors and electronics, can be played in a silent mode (for home rehearsals), but also aloud via an amplifier (at a concert). The main goal is to find a relatively easy and quick way of testing the acoustic performance of various materials and the effect of 3D printing parameters to test such combination at the very beginning of the production process, to minimize the risk of unnecessary costs downstream, if such testing is not performed.

## 2. Materials and Methods

This section describes the materials and methods used in the study.

### 2.1. Materials

The testing specimens were prepared from polyethylene terephthalate with glycol modification (PET-G), acrylonitrile styrene acrylate (ASA), and polylactic acid (PLA). The materials were provided by Prusa Polymers (Prusa Polymers Corp., Prague, Czech Republic) in the form of filaments with a thickness of 1.75 mm. Though PLA is arguably the most popular printing material for the FDM technique due to its user-friendly properties (low melting temperature, good adhesion to the printing bed, low shrinkage upon cooling, and thus, low warp), these qualities unfortunately result in poor mechanical properties. The focus of this paper is thus on the PET-G and ASA filaments, which proved to withstand the load acting on the main parts of the musical instruments. 

### 2.2. Samples’ Production

The samples were produced with a Prusa Mk3S+ printer (Prusa Research Corp., Prague, Czech Republic), which uses a typical Cartesian coordinate printhead (extruder) motion. The PET-G and ASA samples were printed with 230 °C and 260 °C hot-end temperatures, respectively, with a 0.4 mm nozzle diameter. The printing speed was 45 m/s^−1^ for the perimeters and 80 m/s^−1^ for the infill. The samples were also prepared with different infill densities (100% and 20%). The infill density, in simple terms, relates to the free space between neighboring deposited filaments within the shell of the printed object. The infill density of 100% means there are no air gaps between adjacent filaments. The shell is the outer wall of the printed object that outlines the desired shape of the printed structure and provides an anchor for the infill deposition. Infill density influences the mechanical strength of the printed product (the higher the infill, the higher the mechanical strength) and the printing time (the higher the infill, the longer the printing time). Usually, the optimal value is found experimentally. The bed temperature (i.e., the temperature of the platform where the object is 3D-printed) was set to 85 °C for PET-G and ASA and to 110 °C for PLA. The pattern was printed with an inner shell speed of 25 mm/s, outer shell speed of 25 mm/s, and an infill speed of 80 mm/s. 

**Figure 2:**
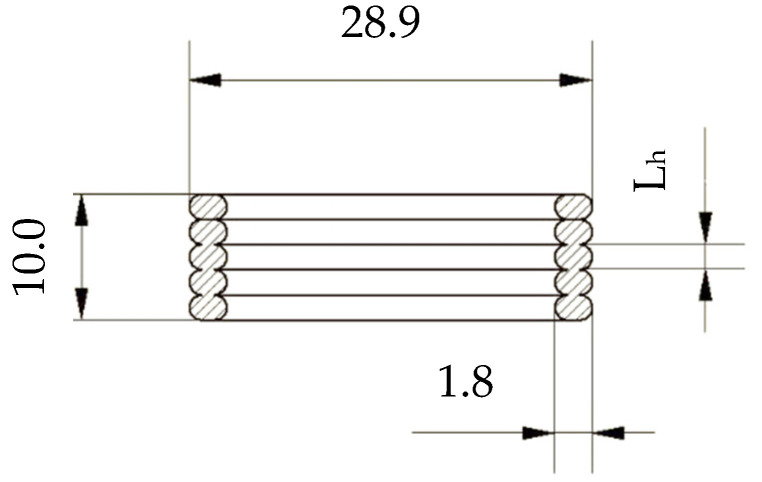
Sample dimensions.

**Figure 3:**
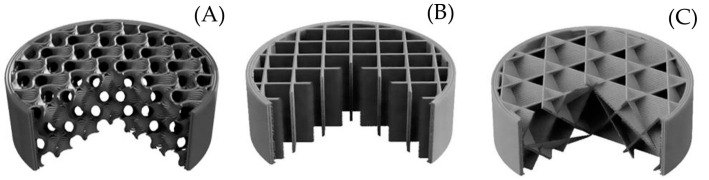
Infill structures: (**A**) Gyroid, (**B**) Grid, and (**C**) Cubic.

In order to evaluate the effect of the surface quality (texture), samples were made with different infill types and layer heights (L_h_), as shown in the Figure 2. The most popular infill types (Gyroid, Grid, and Cubic) were utilized (Figure 3). The surface quality strongly depends on the layer height, which was set to 0.1 mm, 0.3 mm, and 0.5 mm for each material and infill type. The volume ratio, *V_r_*, of porous materials is defined by the equation:

(1) $$V_{r}\left(\%\right)=\frac{V_{S}}{V_{T}}*100$$

where *V_S_* is the volume of solid phase and *V_T_* is the total body volume.

The volume ratio was the same, and therefore samples from different materials differed in their weights. The weight of the PET-G and ASA samples was (3.3 ± 0.1) g and (2.8 ± 0.1) g, respectively. For these reasons, the 3D-printed PET-G samples were characterized by a higher mechanical stiffness compared to the ASA samples.

The sample dimensions are given by the inner dimensions of the testing apparatus. It is important to keep the dimensions as accurate as possible to avoid artifacts resulting from the voids between the sample and the walls of the impedance tube. The dimensions of the samples were 28.9 mm in diameter and 10.0 mm in height. Both values are in the tolerance of ±0.1 mm. As mentioned before, the infill structure of each sample was made with a given structure (Gyroid, Grid, or Cubic), with a volume ratio *V_r_* = 40%, while the shell thickness was 1.8 mm. 

### 2.3. Measurement Methodology

#### 2.3.1. Sound Absorption Coefficient

**Figure 4:**
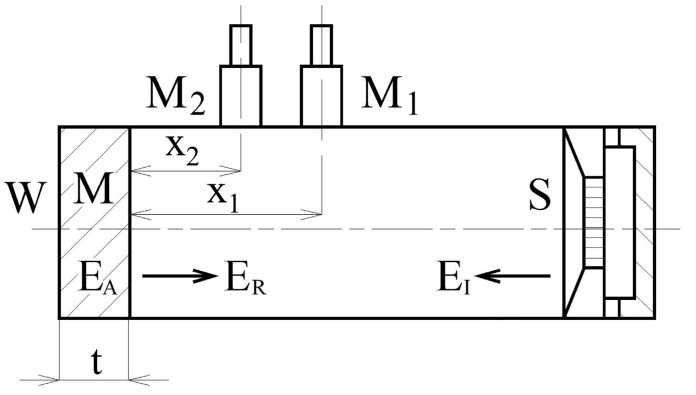
Schematic of the acoustic impedance tube. Legend to the symbols: M—measured sample; M_1_, M_2_—measuring microphones; S—sound source; t—sample thickness; W—solid wall—ideal sound reflection; x_1_, x_2_—microphone distances from the tested sample surface.

Frequency dependencies of the normal incidence absorption coefficient of the investigated samples were measured using a two-microphone acoustic impedance tube (BK 4206), in combination with a signal multi-analyzer PULSE (BK 3560-B-030) and a power amplifier (BK 2706) in the frequency range of 200 to 3200 Hz (all from Brüel & Kjaer, Naerum, Denmark). Each sample was measured three times, and mean values were taken into consideration. The schematic of the acoustic impedance tube is shown in Figure 4.

As the acoustic energy, E_I_, propagates from a noise source to a material surface, a part of the incident acoustic energy is reflected, and the other part is absorbed by the material. The material’s ability to dampen sound is expressed by the sound absorption coefficient, *α*, which is defined by the formula:

(2) $$\alpha =\frac{E_{A}}{E_{I}}=1-\frac{E_{R}}{E_{I}}$$

where *E_A_* represents the absorbed acoustic energy and *E_R_* is the reflected acoustic energy. The ability of sound-absorbing materials is to transform the incident acoustic energy into heat.

Frequency dependencies of the sound absorption coefficient were obtained by the transfer function method ISO 10534-2, which is based on the partial standing wave principle [28]. It is expressed by Equation (3) [29,30]:

(3) $$\alpha =1-{\left|r\right|}^{2}=1-r_{r}^{2}-r_{i}^{2}$$

where *r* is the normal incidence reflection factor, and *r_r_* and *r_i_* are the real and imaginary components of the factor *r*, which is given by [23]:

(4) $$r=r_{r}+ir_{i}=\frac{H_{12}-H_{I}}{H_{R}-H_{12}}\cdot e^{2k_{0}\cdot x_{1}i}$$

where *H*_12_ is the complex acoustic transfer function, *H_I_* is the transfer function for the incident wave, *H_R_* is the transfer function for the reflection wave, *k*_0_ is the wave number, and *x*_1_ is the distance between the investigated material sample and the microphone *M*_1_. The transfer functions are expressed as follows:

(5) $$H_{12}=\frac{p_{2}}{p_{1}}=\frac{e^{k_{0}\cdot x_{2}i}+r\cdot e^{-k_{0}\cdot x_{2}i}}{e^{k_{0}\cdot x_{1}i}+r\cdot e^{k_{0}\cdot x_{1}i}}$$

(6) $$H_{I}=e^{-k_{0}\left(x_{1}\cdot x_{2}\right)i}$$

(7) $$H_{R}=e^{k_{0}\left(x_{1}\cdot x_{2}\right)i}$$

where *p*_1_ and *p*_2_ are the complex acoustic pressures at the two microphone positions, and *x*_2_ is the distance between the investigated material sample and the microphone *M*_2_.

#### 2.3.2. Noise Reduction Coefficient

The ability to absorb sound depends on several factors, such as the thickness of the object, the density of the material, the structure, the excitation frequency, and the temperature. 

The noise reduction coefficient (*NRC*) is expressed as a single number that is defined as the arithmetical average of the sound absorption coefficients at the frequencies 250, 500, 1000, and 2000 Hz, obtained from the impedance tube test [23].

(8) $$NRC=\frac{\alpha_{250}+\alpha_{500}+\alpha_{1000}+\alpha_{2000}}{4}$$

#### 2.3.3. Sound Reflection Coefficient

The sound reflection coefficient (*β*) is calculated from the measured sound absorption coefficient, *α,* as follows:

(9) $$\beta =\frac{E_{R}}{E_{I}}=1-\frac{E_{A}}{E_{I}}=1-\alpha$$

In order to compare the sound reflection properties of the investigated samples, the arithmetic mean of the sound reflection coefficient (β_m_) over the frequency domain (i.e., 200–3000 Hz) was used.

#### 2.3.4. Surface Quality

The produced samples were analyzed with an optical surface profiler, Zygo NewView8000 (Zygo Corporation, Middlefield, CT, USA), which provides non-contact optical surface profiling of materials. This system is nondestructive and requires no sample preparation.

**Figure 5:**
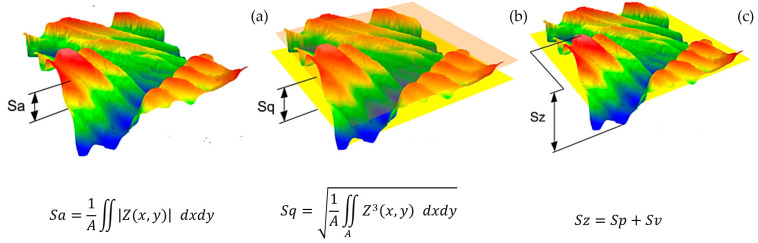
Surface parameters: (**a**) Average roughness evaluated over the complete 3D surface, (**b**) root mean square roughness evaluated over the complete 3D surface, and (**c**) maximum height of the areal surface.

Basic areal surface roughness parameters were used to assess the surface quality of the 3D-printed samples. Average roughness evaluated over the complete 3D surface is shown in Figure 5 as Sa, and its value is described in the equation. The second parameter, Sq, is defined as the root mean square roughness evaluated over the complete 3D surface. The third parameter, Sz, shows the maximum height of the areal surface, where Sp is the maximum peak height of the areal surface and Sv is the maximum valley depth of the areal surface. The complete description of the surface roughness parameters can be found elsewhere [31,32,33,34,35,36].

For the purpose of this paper, one can see Sa and Sz as the areal analogies of the Ra and Rz (arithmetical mean roughness and total profile height, respectively) profile parameters. The *Sdr* parameter, also called the area factor, falls among the so-called hybrid parameters. It represents the ratio between the interfacial and the projected surface area (Equation (10)).

(10) $$Sdr=\frac{\left(Textured\;surface\;area\right)-\left(Cross\;sectional\;area\right)}{\left(Cross\;sectional\;area\right)}$$

## 3. Results and Discussion

This section explores the effects of the materials and the combination of infill structure and surface quality that influence the sound reflection coefficient of the polyethylene terephthalate with glycol modification (PET-G) and acrylonitrile styrene acrylate (ASA).

### 3.1. Frequency Dependencies of the Sound Reflection Coefficient

#### 3.1.1. Effect of Material Type

The choice of material used for a specific application is an important factor that has a strong influence on the sound reflection performance. This effect was tested inside the acoustic impedance tube on samples with 100% infill (zero volume of voids). 

**Figure 6:**
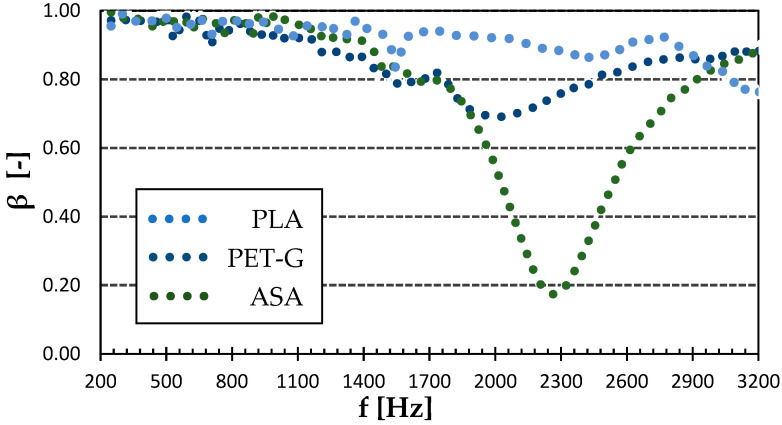
Effect of the material type on sound reflection properties. L_h_ = 0.3 mm.

Figure 6 shows the frequency dependencies of the sound reflection coefficient of selected materials. It is obvious that both examined materials behaved similarly at low-excitation frequencies, namely below 2 kHz. However, the biggest difference was that the ASA sample exhibited the maximum value of the sound absorption coefficient around the excitation frequency of 2.2 kHz. This makes it specific for acoustical application. On the other hand, the ASA sample was characterized by a slightly lower reflection ability at higher excitation frequencies. The sound reflection coefficient of the PLA sample was quite high at lower excitation frequencies, with the first sound reflection peak at around 1.5 kHz. The PLA material unfortunately did not exhibit sufficient mechanical properties to withstand the stresses to which musical instrument parts are subjected, and it was therefore excluded from further investigation. It is obvious (see Figure 6) that lower sound reflection properties were generally found in the case of the ASA specimen, especially at higher excitation frequencies. This is due to the lower mechanical stiffness of this material compared to that of PET-G.

#### 3.1.2. Effect of Layer Height and Infill Structure of PET-G

**Figure 7:**
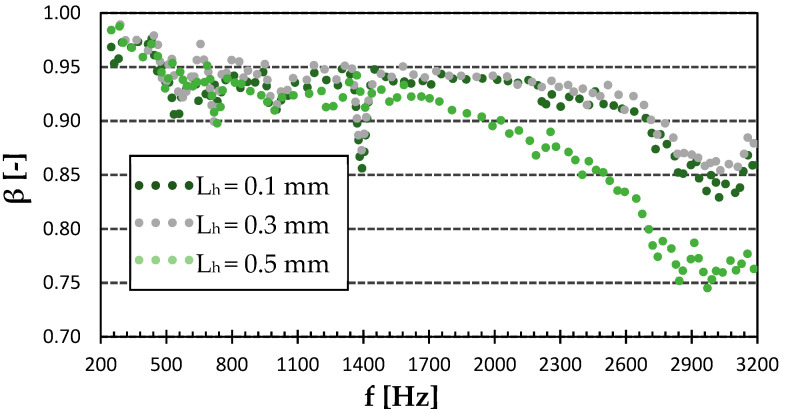
Effect of layer height on frequency dependencies of the sound reflection coefficient (*β*) in the Gyroid infill type of PET-G.

As can be seen in Figure 7, the sound reflection coefficient of the Gyroid structure sample did not depend much on the surface quality. It exhibited very little difference under 2 kHz. However, the lowest surface quality of the 0.5 mm layer height had the lowest sound reflection coefficient at higher excitation frequencies (i.e., f > 1.4 kHz). The smoothest surface with a layer height of 0.1 mm was not necessarily the best in sound reflectivity. It can be seen (see Figure 7) that the sound reflection properties of the smoothest sample were slightly lower compared to the sample produced with a layer height of 0.3 mm at higher excitation frequencies. It is also evident that all tested samples, regardless of their surface quality, exhibited the lowest sound reflectivity at the highest excitation frequencies. It can be concluded that the lowest ability to reflect sound was generally found for the PET-G sample with the Gyroid structure that was printed with a layer height of 0.5 mm. This phenomenon is caused by the higher surface irregularities (i.e., roughness) of this sample, which were accompanied by multiple reflections of incident acoustic waves on these surfaces, and thus a higher transformation of acoustic energy into heat.

**Figure 8:**
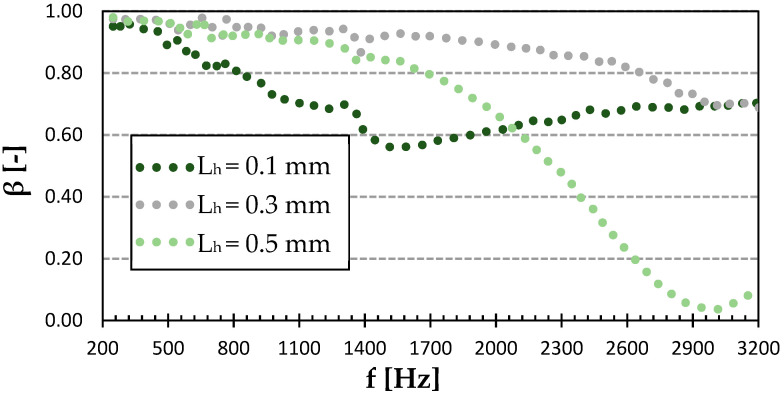
Effect of layer height on frequency dependencies of the sound reflection coefficient (*β*) in the Cubic infill type of PET-G.

Figure 8 shows the sound reflection properties of the Cubic structure printed with a different surface quality. Compared to the rest of the structures, this one had the highest sound-absorbing potential. This was due to the more complex pore shapes of this structure (see Figure 3), which were accompanied by multiple reflections of acoustic waves during their propagation through this material structure and higher dissipation of acoustic energy into heat. For the 0.1 mm layer height, there was a significant minimum in sound reflection at 1.5 kHz, followed by a slow rise towards higher frequencies. The Cubic structure with the 0.5 mm layer height exhibited the lowest sound reflection coefficient at about 3 kHz. The last curve of the 0.3 mm layer height slowly dropped within the studied frequency range to match the performance of the 0.1 mm layer height above 2.9 kHz. It is evident from Figure 8 that the Cubic structure printed with a layer height of 0.3 mm was characterized by the highest ability to reflect sound, which led to a reduction in printing time compared to the sample printed with a layer height of 0.1 mm. However, its sound reflection properties were lower compared to the commonly used wooden materials for the production of musical instruments. For this reason, this 3D-printed material structure is not suitable for use in musical acoustics.

**Figure 9:**
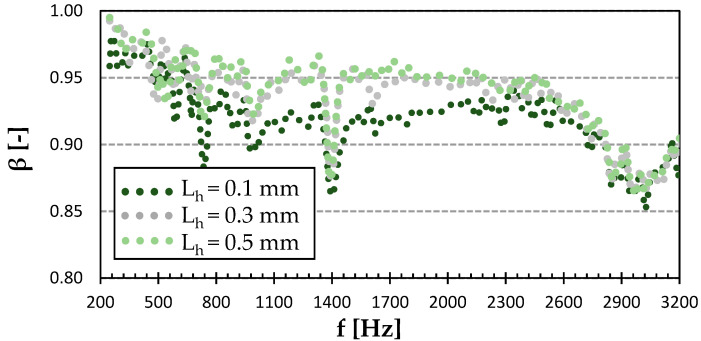
Effect of layer height on frequency dependencies of the sound reflection coefficient (*β*) in the Grid infill type of PET-G.

Figure 9 expands this approach from the Cubic to the Grid structure and shows that the lower surface quality in this infill structure did not significantly increase the sound absorption properties. Thus, the combination of this infill type with a 0.5 mm layer height provided as much as a 60% printing time reduction compared to the conventional 0.2 mm layer height settings, without compromising the expected acoustic performance.

#### 3.1.3. Effect of Layer Height and Infill Structure of ASA

**Figure 10:**
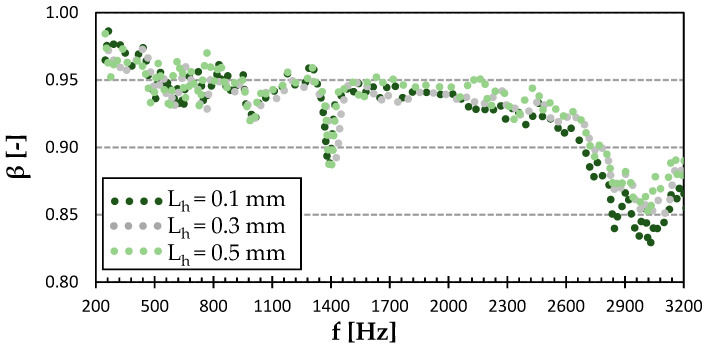
Effect of layer height on frequency dependencies of the sound reflection coefficient (*β*) in the Gyroid infill type of ASA.

As can be seen in Figure 10, the sound absorption properties of the investigated ASA specimens with the Gyroid structure were not significantly affected by the layer height (i.e., the surface quality), as all the curves followed a very similar pattern over the whole frequency range. The frequency dependence for the 0.1 mm layer height was characterized by a slightly higher sound absorption compared to other layer heights (i.e., 0.3 and 0.5 mm), but the difference was negligible. 

**Figure 11:**
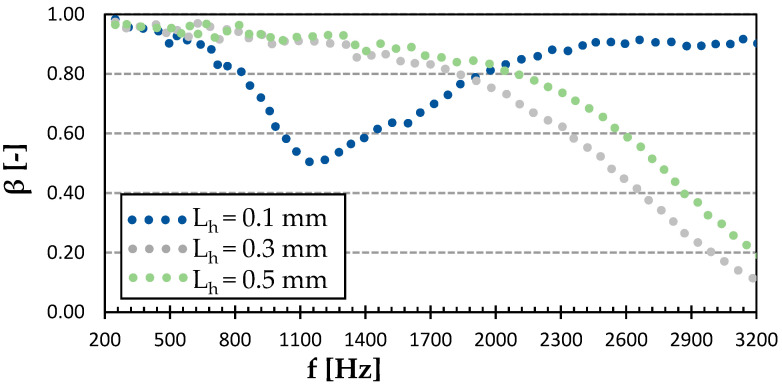
Effect of layer height on frequency dependencies of the sound reflection coefficient (*β*) in the Cubic infill type of ASA.

As revealed by the graph in Figure 11, a significant effect of the surface quality on the sound reflection properties was observed for the Cubic infill type. The sample printed with a layer height of 0.1 mm absorbed more sound in the frequency range from 0.8 to 1.7 kHz, while reflecting most of the incident acoustic energy in the rest of the frequency range. Both samples with higher layer heights (i.e., 0.3 and 0.5 mm) followed a very similar pattern of a continuously decreasing sound reflection with the increasing frequency, with a steeper decline from 2000 Hz onwards, where the sample printed with a layer height of 0.3 mm exhibited slightly higher sound absorption (as also shown by the NRC value in Table 1). Similarly, as in the case of the PET-G samples produced with the Cubic structure, it can be stated that the investigated 3D-printed ASA samples with the same material structure were not suitable to reflect sound.

**Figure 12:**
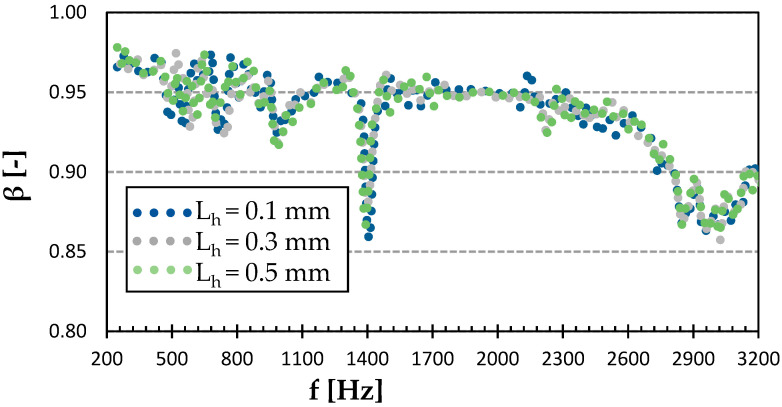
Effect of layer height on frequency dependencies of the sound reflection coefficient (*β*) in the Grid infill type of ASA.

Figure 12 shows the effect of surface quality on the sound reflection properties of the Grid infill structure. Again, similar to PET-G samples with the same structure, no significant difference was apparent. Therefore, with a layer height of 0.5 mm, the same acoustic performance could be achieved, with a very noticeable 3D printing time reduction. It also appears that there was very little difference in the overall acoustical performance between the ASA material printed with the Gyroid and Grid structures, except for the more significant drop at the frequency of 1.4 kHz for the Grid structure (Figure 10 and Figure 12). 

### 3.2. Noise Reduction Coefficient and Average Sound Reflection Coefficient

As mentioned above, the noise reduction coefficient (NRC) is used to describe the average sound absorption performance of a given material. The average sound reflection coefficient, β_m_, uses a different approach and compares the samples by the arithmetic mean of the sound reflection coefficient, β, over the evaluated frequency domain. The closer the value is to 1, the better the tested sample’s ability to reflect sound.

As can be seen in Table 1, the NRC of the 100% infill samples was 0.119 and 0.139 for the PET-G and ASA materials, respectively. In the case of the 100% PLA infill sample, the value of this coefficient was NRC = 0.051, from the measured frequency dependence of the sound absorption coefficient of this material sample (see Figure 6). From this perspective, the 3D-printed ASA material samples exhibited a higher noise absorption potential compared to the other investigated materials, which can be expected from the position of its curve in the vertical direction in Figure 6. The behavior at lower excitation frequencies is the most important for producing musical instruments.

It is evident from Table 1 that the lowest sound reflection properties, independently of the layer height, were found for the ASA and PET-G specimens manufactured with the Cubic structure. Low values of the average sound reflection coefficient of this material structure corresponded to higher values of the NRC. As mentioned above, the Cubic structure is characterized by a complex shaped material structure, in which the propagation of acoustic waves results in multiple sound reflections within the structure. The consequence of these multiple sound reflections is a higher sound absorption, and thus a lower ability of these material structures to reflect sound. For these reasons, the 3D-printed samples produced with the Cubic structure are not suitable materials in musical acoustics. In contrast, the best sound reflection properties were observed for the ASA and PET-G specimens made with the Grid structure, regardless of the layer height. This is due to the relatively simple lattice structure of these samples (see Figure 3), which creates a low acoustic resistance relative to the incident acoustic waves in the acoustic impedance tube (see Figure 4). Therefore, the investigated 3D-printed specimens produced with the Grid structure were characterized by very low sound absorption properties and can be perspective materials to produce musical instruments. Another indisputable advantage of these sound-reflecting materials is the reduction of their density, and thus the weight of the musical instruments produced. As is shown in Table 1, slightly higher values of NRC were observed for samples fabricated with the Gyroid structure compared to the Grid material structure. For this reason, the Gyroid infill type is also suitable to reflect sound.

The best sound reflection performance was achieved using the PET-G sample with the Grid infill structure of the 0.3 mm layer height, which is currently a good result providing sufficient acoustic properties in relatively fast production. However, the same material with the same infill structure and with a higher layer height, L_h_ = 0.5 mm (lower surface quality), still exhibited a quite high β_m_ coefficient, and further offers a significant time reduction (especially important in serial or mass production).

Many researchers have investigated the sound absorption properties of different types of wood with similar sample thicknesses (i.e., *t* ≅ 10 mm). Basic raw, treated, and perforated woods were investigated in different studies. Taghiyari et al. [37] studied the sound absorption properties of four hardwoods and one softwood, namely, beech, poplar, walnut, white mulberry, and fir, at four different excitation frequencies (i.e., 800, 1000, 2000, and 4000 Hz). In addition, mulberry and walnut were chosen as they are traditionally used for musical instruments in Iran. The wood samples were manufactured both in the longitudinal and tangential fiber directions. It was found in this work that the lowest sound absorption properties (i.e., α_min_ = 0.08) were obtained for beech, walnut, and mulberry wood specimens with a tangential fiber direction at the frequency of 800 Hz. Higher sound absorption properties were obtained at higher excitation frequencies, independently of the wood type. In general, the sound reflection performance of the above-mentioned wood specimens was poorer compared to the investigated porous polymer (i.e., ASA and PET-G) material samples. Kang et al. [38] investigated the sound-damping properties of three dry types of Japanese wood, namely Sugi, Chanchin-modoki, and Yurinoki woods. Furthermore, sapwood and heartwood samples from each of the three wood types were examined. It was found that the NRC was from 0.04 (i.e., Sugi and Chanchin-modoki heartwood) to 0.14 (i.e., Yurinoki sapwood). A comparison of the sound absorption properties of chinaberry and ginkgo wood samples, namely control and steam-exploded (i.e., treated) samples, was evaluated in [39]. It was found in this work that the treated wood samples exhibited better sound absorption properties compared to the control samples, independently of the wood type. It can be stated that the ability to reflect sound of the chinaberry and ginkgo wood samples was worse compared to the polymer specimens, which were investigated in this work. Xu et al. [40] studied the effect of the coating thickness (i.e., from 0 to 0.6 mm) of four different types of spruce woods on their sound absorption behavior in the frequency range from 125 to 2000 Hz. In addition, these wood types are commonly used for piano soundboards. It was found in this work that the sound absorption properties of the spruce woods generally increased with the increasing coating thickness. The perforating effect on the sound absorbance of panels, which were made of Mongolian Scotch pine, was investigated by Song et al. [41]. It was established that the ability to dampen sound generally increased with the increasing perforation diameter. Therefore, these perforated wood panels are not suitable to reflect sound compared to the 3D-printed specimens investigated in this work. 

It is clear from the above that the investigated 3D-printed ASA and PET-G polymer samples made with the Grid and Gyroid infill structures are promising materials in the production of musical instruments in order to improve sound quality, compared to the commonly used wooden materials.

### 3.3. Surface Quality Analysis

**Figure 13:**
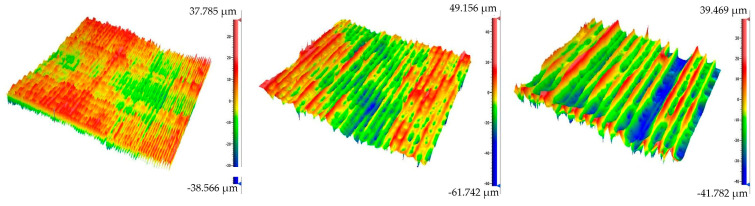
The micrographs of the surface texture affected by the printing parameters’ setting: 0.1 mm layer height (**left**), 0.3 mm layer height (**middle**), and 0.5 mm layer height (**right**).

As mentioned above, Zygo NewView 8000 was used to analyze the surface texture. Based on the previous experience and new acoustical data (Figure 6), the PET-G material seems to have the highest potential for the production of stringed musical instruments with the FDM method; therefore, the surface analysis of this material was performed. The micrographs are presented in Figure 13.

The quality of the PET-G surface is described in Table 2. It compares several surface parameters, such as Sa, Sq, Sz, and Sdr. 

As can be seen in Table 2, there was a very small difference in the Sa and Sq parameters between the 0.1 mm and 0.3 mm layer heights. There was, however, an almost 40% difference in these values when comparing the 0.3 mm and 0.5 mm layer heights. Evidently, the first-layer effects (the deformation of the first layer in the 3D printing process) play some role here. Interestingly, from the point of view of the maximum height (Sz parameter), the 0.3 mm layer height sample exhibited a higher surface texture. One can thus conclude that from this perspective, it is wise to opt for the 0.5 mm printing setting as it provides a comparable tactile response to the finer settings, with a much lower production time. In addition, there are studies confirming the better mechanical properties of samples printed with thicker layers [42]. The Sdr parameter would suggest better sound-damping properties of the 0.1 mm layer samples, but this was not experimentally proven. This parameter and the effect of the specific surface thus deserve further investigation. 

## 4. Conclusions

This study has investigated the sound reflection performance of selected 3D-printed materials at different excitation frequencies. Additive manufacturing (3D printing) is a fast option in production compared to conventional methods; however, the manufacturing time is highly dependent on the process parameters. The most dominant factors in printing time are the layer height and infill density. The increase in the layer height (which significantly reduces the printing time) is usually accompanied by higher surface texture (high surface roughness). However, the data from the surface analysis suggest that even though the Sa and Sq parameters could be affected by the layer height setting, there was very little difference between the surface quality printed at 0.1 mm and 0.3 mm layer heights. In addition, the samples printed with the 0.5 mm layer height resulted in a surface with an Sz value (total profile height) lower than that of the 0.3 mm layer height sample, and very close to the 0.1 mm layer height sample. This suggests that opting for the higher layer height can be a reasonable step, as with low layer height settings the printing time significantly increases and can make the whole process economically unsustainable. An increased layer height also goes hand-in-hand with the improvement of mechanical properties. From the acoustical point of view, the layer height does not play a very significant role, except for the Cubic infill geometry. Here, significant variations of the sound reflection coefficient with the layer height were found. As the outcome is quite unpredictable, the suggestion is to avoid this structure in 3D-printed musical instruments. Concerning the material selection itself, the acoustical data alone would be in favor of PLA. Unfortunately, this material (often a first choice in entry-level 3D printing) is known for its poor mechanical properties, and it would not withstand the stress caused by the pull of the strings in the musical instruments. 

Summarizing the relevant data from our recent preliminary study and taking into account the point of view of acoustics, as well as the economic constraints, the ideal combination for the 3D printing of string instruments seems to be the PET-G material with either a Gyroid or Grid infill structure, printed with a 0.3 or 0.5 mm layer height. This is not to say that other combinations are not possible—printing with more advance materials, such as carbon fiber-reinforced Nylon, would result in high-strength prints with good acoustical performance, but the additional cost (material price itself and prolonged printing time) would possibly render the whole process economically unviable. Further research will thus be focused on selectively applying such advanced materials, in the most demanding locations of the instrument body. The combination of plastic with metal reinforcement and its application potential will also be studied. Furthermore, the effort will be focused on finding alternative ways of testing the acoustical properties of materials, such as vibration and resonance analysis, as well as testing pre-production samples of the 3D-printed instruments. 

## Figures and Tables

**Table 1:** Measured values of the noise reduction coefficient and the average sound reflection coefficient of each infill structure for various layer heights and material types.

		NRC [-]		β_m_ [-]	
L_h_ (mm)	Infill Type	ASA	PET-G	ASA	PET-G
0.1	Gyroid	0.055	0.072	0.925	0.919
0.1	Cubic	0.163	0.193	0.799	0.705
0.1	Grid	0.054	0.059	0.936	0.919
0.3	Gyroid	0.057	0.048	0.928	0.929
0.3	Cubic	0.109	0.060	0.703	0.878
0.3	Grid	0.052	0.041	0.936	0.963
0.5	Gyroid	0.058	0.064	0.933	0.886
0.5	Cubic	0.092	0.124	0.766	0.632
0.5	Grid	0.055	0.048	0.936	0.940
0.2	100% infill	0.139	0.119	0.758	0.852

**Table 2:** Areal roughness parameters of the studied PET-G samples.

Layer Height (mm)	Sa (µm)	Sq (µm)	Sz (µm)	Sdr (-)
0.1	10.1	12.7	76.4	0.19
0.3	9.7	12.1	110.9	0.02
0.5	13.9	16.5	81.3	0.02

## Data Availability

The data presented in this study are available on request from the corresponding author.
